# Short Symmetric-End Antimicrobial Peptides Centered on β-Turn Amino Acids Unit Improve Selectivity and Stability

**DOI:** 10.3389/fmicb.2018.02832

**Published:** 2018-11-27

**Authors:** Na Dong, Shuli Chou, Jiawei Li, Chenyu Xue, Xinran Li, Baojing Cheng, Anshan Shan, Li Xu

**Affiliations:** The Laboratory of Molecular Nutrition and Immunity, Institute of Animal Nutrition, Northeast Agricultural University, Harbin, China

**Keywords:** antimicrobial peptide, cell selectivity, condition-resistance, bactericidal mechanism, hemolysis

## Abstract

Antimicrobial peptides (AMPs) are excellent candidates to combat the increasing number of multi- or pan-resistant pathogens worldwide based on their mechanism of action, which is different from that of antibiotics. In this study, we designed short peptides by fusing an α-helix and β-turn sequence-motif in a symmetric-end template to promote the higher cell selectivity, antibacterial activity and salt-resistance of these structures. The results showed that the designed peptides PQ and PP tended to form an α-helical structure upon interacting with a membrane-mimicking environment. They displayed high cell selectivity toward bacterial cells over eukaryotic cells. Their activities were mostly maintained in the presence of different conditions (salts, serum, heat, and pH), which indicated their stability *in vivo*. Fluorescence spectroscopy and electron microscopy analyses indicated that PP and PQ killed bacterial cells through membrane pore formation, thereby damaging membrane integrity. This study revealed the potential application of these designed peptides as new candidate antimicrobial agents.

## Introduction

Antimicrobial peptides (AMPs) are widespread in nature; are produced by a variety of tissues in invertebrate, plants, and animal species; they play an important role in non-specific interactions against invading pathogenic microorganisms, including bacteria, viruses, parasites and even cancer cells (Andersen et al., 2004; Papo and Shai, 2005; Migliolo et al., 2016). AMPs act at the cytoplasmic membrane leading to permeabilization and eventual membrane disruption, which are different from the actions of antibiotics and are less likely to promote the development of resistant bacteria (Hancock and Sahl, 2006; Rai et al., 2016). Despite the advantage of AMPs, the major drawback of naturally occurring AMPs, such as PG-1 or melittin, is their cytotoxicity. Many novel synthetic peptides developed based on these naturally occurring AMPs have been reported with similar antimicrobial activities but reduced cytotoxicity (Montesinos, 2007; Datta et al., 2016). Over decades of research, it has become clear that the antimicrobial activity and selectivity of peptides toward microbial cells depends on amphipathicity, hydrophobicity, charge, length and structure (Findlay et al., 2010; Costa et al., 2011). Amphipathicity favors peptide internalization and in turn membrane perturbation, hydrophobicity leads to strong partitioning into membranes, positive charge is essential for initial binding to the negatively charged bacterial membrane surface, and peptide structure is crucial for binding to target membranes (Malanovic and Lohner, 2016; Mura et al., 2016).

Antimicrobial peptides have a range of different secondary structures, such as α-helix, β-sheet, or β-hairpin, and extended or loop-like structures (Jenssen et al., 2006). The α-helical AMPs are particularly successful structural arrangements in innate defense, and amphipathic β-hairpin AMPs exhibit high cell selectivity (Auvynet and Rosenstein, 2009; Dong et al., 2012; Chou et al., 2016). It has been suggested that peptide antimicrobial activity initially increases and then decreases with chain length and that longer peptides may stimulate more toxicity in mammalian cells. In contrast, short AMPs are exceptionally well suited because they can be synthesized quickly and easily, modified and optimized with less cost, and they may not trigger an undesired immune response (Carotenuto et al., 2008; Grieco et al., 2013a,b; Bagheri et al., 2016; Mikut et al., 2016).

We have recently reported on symmetric-end peptides based on a β-hairpin structure that have a broad spectrum of antimicrobial activity and high cell selectivity (Dong et al., 2014). The characterization of symmetric-end peptides and multiple-stranded β-hairpin peptides, both of which have broad-spectrum antimicrobial activity and high cell selectivity, leads us to propose that a symmetrical method may improve the cell selectivity of a peptide with high antimicrobial activity. Other studies have indicated that the α-helical conformation plays a crucial role in determining AMP activity (Huang, 2000), as the enhanced helical stability at the membrane interface is a key driver for increased efficacy (Dennison and Phoenix, 2011). To combine the advantages of these structures, we designed novel symmetric-end AMPs by embedding a β-turn-promoting sequence within two symmetric α-helical units in this study. The α-helical unit is composed of different types of hydrophobic amino acids including Ile (I), Phe (F), Trp (W), and positively charged amino acids including His (H), Lys (K), and Arg (R). There are four types of motifs in these newly designed peptides including “Pro (P), Gly (G)”; “Cys (C), Arg (R), Arg (R), Arg (R), Phe (F), Cys (C)”; “Gly (G), Gly (G)”; or none. All peptides were amidated at the C-terminus. The secondary structures of these peptides were characterized by circular dichroism (CD) in membrane-mimicking environments and in aqueous solution. Antimicrobial activities in different conditions (including salts, serum, heat, and pH) were examined using minimum inhibitory concentration (MIC) measurements. Hemolytic activity and cytotoxicity were also determined, and the peptide-membrane interactions were evaluated using fluorescence and scanning electron microscopy assays. We also propose a novel peptide-based antimicrobial agent heterozygous model to generate effective AMPs with great antimicrobial activities and cell selectivity.

## Materials and Methods

### Materials

Test strains including Gram-negative and Gram-positive bacteria were adopted as follows. Gram-negative bacteria were *Escherichia coli* ATCC 25922, *E. coli* UB 1005, *Bacterium pyocyaneum* ATCC 27853, *Salmonella pullorum* C79-13, and *Salmonella entericaserovar Typhimurium* C77-31. Gram-positive bacteria were *Staphylococcus aureus* ATCC 29213, *Staphylococcus aureus* ATCC 43300, *Staphylococcus epidermidis* ATCC 12228, *Streptococcus faecalis* ATCC 29212, and *Bacillus subtilis* CMCC 63501. The above bacteria were obtained from the School of Veterinary Medicine, Northeast Agricultural University (Harbin, China). *E. coli* UB 1005 was kindly provided by Prof. Q. S. Qi (State Key Laboratory of Microbial Technology, Shandong University, China). Red blood cells (RBCs) used in the experiments were extracted from healthy blood donors. Intestinal epithelial cells (IPEC-J2) were donated by the Northeast Agricultural University (Harbin, China).

Mueller–Hinton broth (MHB) powder and Mueller–Hinton Agar (MHA) powder were obtained from AoBoX (China). Bovine serum albumin (BSA) and sodium dodecyl sulfate (SDS) were obtained from Sigma-Aldrich (China), and trifluoroethanol (TFE) was purchased from Amresco (United States). Phosphate-buffered saline solution was obtained from Kermel (China). MTT (3-(4,5- Dimethylthiazol-2-yl)-2,5-diphenyltetrazolium bromide) was purchased from Sigma-Aldrich Corporation (China), and DMEM supplemented with L-glutamine and 10% fetal calf serum was obtained from Invitrogen Corporation (United States). Sodium chloride, potassium chloride, ammonium chloride, zinc chloride, magnesium chloride, and ferric chloride were all analytical grade and purchased from Kermel (China). Triton X-100, N-phenyl-1-naphthylamine (NPN), 3,3^′^-dipropylthiadicarbocyanine iodide (diSC3-5), HEPES and ethylene diamine tetraacetic acid (EDTA) were purchased from Sigma-Aldrich (China). 1-palmitoyl-2-oleoyl-*sn-*glycero-3-[phospho-rac-(1-glycerol)] (sodium salt) (POPG, Avestin, Inc., Canada) was used in the liposome leakage assay.

All peptides were synthesized and analyzed by GL Biochem (Shanghai, China). These peptides’ fidelities were identified via matrix-assisted laser desorption/ionization time-of-flight mass spectrometry (MALDI-TOF MS, Linear Scientific Inc., United States), using α-cyano-4-hydroxycinnamic acid as the matrix. The peptides’ purities were confirmed as higher than 95% with analytical reverse-phase high-performance liquid chromatography (RP-HPLC) (Supplementary Data Sheet S1), and the accurate purities for PP, PQ, GG, Qna, Qa, and PG-1 were 95.28, 95.15, 95.48, 97.02, 98.36, and 97.57%, respectively. Quantitative peptides were dissolved in deionized water and stored at -20°C before the assessments. The rest of the peptides were stored as powders at -20°C to prevent peptide degradation.

### Peptide Design and Structure Analysis

We designed a series of novel symmetric-end AMPs by embedding β-turn-promoting motifs within two symmetric α-helical units. First, the α-helical unit composed of 6 residues (IHKFWR) representing a different structural type with high hydrophobic values and a perfect amphipathic structure was selected. Then, the β-turn sequence-motif was embedded to combine the two α-helical units symmetrically to increase the antimicrobial activity and cell selectivity. The “PG” turn is the usual β-turn motif, while the “CRRRFC” turn, which composes the middle 9–12 residues of PG-1, is a stable β-turn sequence-motif. Two α-helical units combined without amino acids or with “GG” turn were also synthesized. All peptides were synthesized based on their linear form without disulfide bonds. The amino acid sequences of the novel symmetric-end α-helices with or without the β-turn motif AMPs are listed in Table 1.

**Table 1:** Peptide design and key physicochemical parameters.

Peptides	Sequences	Formula	TMW^a^	MMW^b^	Net charge	Retained time	*H*^c^	μHrel^d^
PQ	IHKFWRCRRRFCRWFKHI-NH_2_	C_121_H_180_N_42_O_18_S_2_	2575.13	2575.17	+10	12.385	-0.84	4.22
PP	IHKFWRPGRWFKHI-NH_2_	C_95_H_135_N_29_O_14_	1907.27	1907.32	+7	11.409	0.49	3.01
GG	IHKFWRGGRWFKHI-NH_2_	C_92_H_131_N_29_O_14_	187.22	187.27	+7	10.24	0.33	2.91
Qa	IHKFWRRWFKHI-NH_2_	C_88_H_125_N_27_O_12_	1753.11	1753.15	+7	10.11	0.78	6.09
Qna	IHFKWRRWKFHI-NH_2_	C_88_H_125_N_27_O_12_	1753.11	1753.15	+7	10.027	0.78	1.69
PG-1	RGGRLCYCRRRFCVCVGR-NH_2_	C_88_H_151_N_37_O_19_S_4_	2155.61	2156.35	+7	12.56	-2.55	2.40


*^*a*^TMW, theoretical molecular weight; ^*b*^MMW, measured molecular weight (measured by mass spectroscopy); ^*c*^The mean hydrophobicity (H) is the total hydrophobicity (sum of all residue hydrophobicity indices) divided by the number of residues. ^*d*^The relative hydrophobic moment (μHrel) of a peptide is its hydrophobic moment relative to that of a perfectly amphipathic peptide according to the scale of CCS (Cornette et al., 1987). This gives a better idea of the amphipathicity using different scales*.

Amino acid sequence analysis of the three peptides was performed using the ProtParam bioinformatics program (ExPASy Proteomics Server^1^). The secondary content of the peptides was calculated online with K2D2^2^. The three-dimensional structure projection was predicted with I-TASSER^3^.

### Circular Dichroism (CD) Analysis

The secondary structure of the peptides in different environments was measured by CD using a previously described method (Zhu et al., 2015). CD measurements were performed on a J-820 spectropolarimeter (Jasco, Tokyo, Japan) equipped with a rectangular quartz cell with a path length of 0.1 cm. CD spectra of the peptides were detected in different environments including 10 mM sodium phosphate buffer (pH 7.4), 50% TFE and 30 mM SDS micelles at 25°C, with spectra recorded from 190 to 250 nm at a scanning speed of 10 nm/min. The results from three scans were collected and calculated for each peptide. The final concentration of peptide in each buffer was 150 μM.

The mean residue ellipticity was calculated according to the following equation:

(1) θM=(θobs1000)/(cln)

where θ_M_ is the mean residue ellipticity [deg.cm^2^.dmol^-1^], θ_obs_ is the observed ellipticity corrected for the buffer at a given wavelength [mdeg], *c* is the peptide concentration [mM], l is the path length [mm] and *n* is the number of amino acids.

### Hemolytic Activity

The hemolytic activity of the peptides was detected using a previously described modified method (Wang et al., 2015). One milliliter of fresh hRBCs was obtained from a healthy donor (Zhihua Wang, Harbin, China) in a polycarbonate tube containing heparin. The collected hRBCs were centrifuged at 1000 × *g* for 5 min at 4°C and resuspended in PBS buffer (pH 7.2). The hRBCs were washed three times and then incubated with an equal volume of the respective peptide dissolved in PBS for 1 h at 37°C. Then, the hRBC suspension was placed into a 96-well microtiter plater with 50 μL of tested peptide solution at different concentrations and the mixture was incubated for 1 h at 37°C. After incubation, the plate was centrifuged at 1000 × *g* for 5 min and the supernatants were transferred to a new 96-well cell culture plate and examined by measuring the absorbance at 570 nm (OD_570_). hRBCs in PBS and 0.1% Triton X-100 were used as negative and positive controls, respectively. The minimum hemolytic concentrations (MHCs) were defined as the peptide concentrations resulting in 5% hemolysis.

### Cytotoxicity Assay

To determine the cytotoxicity of each peptide on intestinal epithelial cells (IPEC-J2), we used the MTT dye reduction assay, as previously described. Briefly, 1.0–2.0 × 10^5^ cells/well in DMEM (supplemented with 10% heat-inactivated FBS) were placed into 96-well plates and then incubated for 24 h at 37°C in 5% CO_2_. Next, the peptides were added to cell cultures at final concentrations of 0.5–128 μM; untreated cell cultures served as controls the next day. The cell cultures were further incubated for 24 h and then mixed with MTT (50 μL, 0.5 mg/mL). After the mixtures were incubated for 4 h at 37°C, they were centrifuged at 1000 × *g* for 5 min and the supernatants were discarded. Subsequently, 150 μL of DMSO was added to dissolve the formed formazan crystals. Finally, the OD was measured using a microplate reader (TECAN GENios F129004; TECAN, Austria) at 570 nm.

### Antimicrobial Assays

The minimal inhibitory concentration (MIC) was determined according to a previously described method (Chou et al., 2016). Bacteria were cultured overnight to mid-log phase in MHB at 37°C and then diluted to give final concentrations ranging from 2 × 10^5^ to 7 × 10^5^ CFU/mL. Then, 50 μL of serial twofold dilutions of the peptides were added to each well in 96-well microtiter plates with a mixed solution containing 0.01% acetic acid and 0.2% bovine serum albumin (BSA, Sigma) was used to dissolve and dilute the peptides. Then, equal amounts of bacterial aliquots were incubated with the above peptide solution for 18–24 h at 37°C. The experiment was performed in triplicate using three replicates for each peptide and each bacterium. The broth was employed as a negative control and the broth with microbial cells was used as a positive control. The lowest concentration of peptide that prevented visible turbidity was defined as the MIC.

### Time Killing Assay

The ability of peptides to kill bacterial cells was further investigated by analyzing the fraction of cell survival upon peptide treatment at various exposure times according to a previously described method (Wiradharma et al., 2012). Briefly, *E. coli* and *S. aureus* were treated at MIC and 2 × MIC concentrations with peptides. At various time periods (0, 5, 10, 30, 60, 90, 120, and 150 min), microbial suspensions were diluted at three different dilutions and plated on MHA plates. Microbial colonies were formed and counted after 24 h of incubation. The results were presented as the mean data from three independent assays.

### Condition Sensitivity Assays

The condition sensitivity of peptides was tested in an MIC assay in different environments, including salt sensitivity, thermal sensitivity and different pH values similar to our previous method (Maisetta et al., 2008; Ma et al., 2012). For salt sensitivity, *E. coli* ATCC 25922 and *S. aureus* ATCC 29213 were incubated in the presence of different final concentrations of physiological salts (150 mM NaCl, 4.5 mM KCl, 6 μM NH_4_Cl, 8 μM ZnCl_2_, 2 mM CaCl_2_, 1 mM MgCl_2_, and 4 μM FeCl_3_). For the heat resistance experiments, the peptides were incubated at 100°C for 30 min and cooled on ice for 10 min. To test the effects of different pH values on the antimicrobial activity of the peptides, the buffer solution used in this study was adjusted with HCl or NaOH to obtain final pH values of 4.0, 6.0, 8.0, and 10.0. The determination methods for these MIC assays were same as described above.

### Serum Stability Assays

To test the effects of serum on the antimicrobial activities of the peptides, human serum was dissolved in MHB to reach final concentrations of 25 and 50%. The determination methods for these MIC assays were same as described above.

The serum stability of the peptides was further evaluated. First, peptide solution was incubated at 37°C with 50% human serum. Samples withdrawn after 24 h were precipitated with 200 μL of methanol and centrifuged for 1 min at 10,000 × *g*, and then the crude solution was analyzed. HPLC was performed on all samples using a C18 column. The crude solution was diluted in 0.1% trifluoroacetic acid and the UV lamp was set at 280 nm for all detected peptides. The controls for the peptide retention time in the mixture were obtained by adding the same concentrations of peptides to supernatants of serum treated with methanol and centrifuged as above and running the mixture immediately. When HPLC peaks corresponding to uncleaved peptides were no longer detectable after incubation with serum, the HPLC eluent was collected at the appropriate retention time, allowing a window of ± 2 min, and analyzed by MS to assess the absence of uncleaved peptides. LC-MS/MS analysis was performed on a nanoliter liquid system (Eksigent nanoLC-Ultra^TM^, AB SCIEX) and then the samples were scanned with a TripleTOF5600 system (AB SCIEX). The data were analyzed with the Mascot 2.3 software (Matrix Science).

### Antibacterial Mechanism Study

#### Outer Membrane (OM) Permeability Assay

The fluorescent dye NPN was employed to determine the outer membrane permeability using a previously described method (Hein-Kristensen et al., 2013). Briefly, *E. coli* UB 1005 cells in mid-log phase were diluted to 10^5^ CFU/mL in 5 mM sodium HEPES buffer, pH 7.4, containing 5 mM glucose. Each 2 mL of bacteria cells was added to a quartz cuvette with NPN at a final concentration of 10 μM. The background fluorescence was recorded at excitation (λ = 350 nm) and emission (λ = 420 nm) wavelengths. Then, the peptides were added to the cuvette and the fluorescence was recorded until there was no further increase in fluorescence. The fluorescence results were detected with an F-4500 fluorescence spectrophotometer (Hitachi, Japan). The values were converted to % NPN uptake according to the following equation:

(2) $$\%\;\mathrm{NPN}\;\mathrm{uptake}=(F_{\mathrm{obs}}-F_{0})/(F_{100}-F_{0})\times 100$$

where *F*_obs_ is the observed fluorescence at a given peptide concentration, *F*_0_ is the initial fluorescence of NPN with *E. coli* cells in the absence of peptide, and *F*_100_ is the fluorescence of NPN with *E. coli* cells upon addition of 10 μg/mL polymyxin B, which is used as a positive control in this assay.

#### Cytoplasmic Membrane Electrical Potential Measurement

The interaction of the peptides with the bacterial cell cytoplasmic membrane was detected using the membrane potential-sensitive fluorescent dye diSC_3_-5 according to a previously described method (Chou and Fasman, 1978). Briefly, *E. coli* UB1005 cells were cultivated to the mid-log phase at 37°C and diluted to an OD_600_ of 0.05 in buffer (5 mM HEPES and 20 mM glucose, pH 7.4). The bacteria cells were incubated with 0.4 μM diSC_3_-5 until a stable reduction in the fluorescence was achieved. Then, 100 mM KCl was then added to the above cell suspension. A 2 mL cell suspension and the desired concentration of peptide was added to a 1 cm cuvette. The fluorescence change was recorded on an F-4500 fluorescence spectrophotometer (Hitachi, Japan). The maximal increase in the fluorescence was recorded at the desired excitation wavelength (λ = 622 nm) and emission wavelength (λ = 670 nm). The background value was monitored with a blank containing the cells and dye alone.

#### Dye Leakage Assays

Prepared calcein-entrapped large unilamellar vesicles (LUVs) were optimized using a previous method (Stromstedt et al., 2009). The negatively charged lipids composed of POPG/CL (3:1) were dissolved in chloroform, dried with a stream of nitrogen and resuspended in dye buffer solution (70 mM calcein, 10 mM Tris, 150 mM NaCl, and 0.1 mM EDTA, pH 7.4). The suspension was subjected to 10 freeze-thaw cycles in liquid nitrogen and extruded 21 times through polycarbonate filters (two stacked 100-nm pore size filters) with a LiposoFast extruder (Avestin, Inc., Canada). Untrapped calcein was removed by gel filtration on a Sephadex G-50 column. The lipid concentration was determined by quantitative phosphorus analysis. Aliquots of the liposome suspensions were diluted using Tris-HCl buffer to a final concentration of 100 μM lipid and incubated for 15 min with concentrations of peptide solution ranging from 2 to 64 μM. The leakage of calcein from LUVs was monitored by measuring the fluorescence intensity at an excitation wavelength of 490 nm and an emission wavelength of 520 nm on an F-4500 Fluorescence Spectrophotometer (Hitachi, Japan). The maximum dye leakage release was obtained using 0.1% Triton X-100. The percentage of the calcein release caused by the AMPs was calculated using the following equation:

(3) $$\mathrm{Dye}\;\mathrm{release}\;(\%)=(F_{\mathrm{obs}}-F_{0})/(F_{100}-F_{0})\times 100\%$$

where *F*_0_ is the fluorescence intensity of the liposomes (background) and *F*_obs_ and *F*_100_ are the fluorescence intensities achieved by the peptides and Triton X-100, respectively.

#### LPS-Binding Assay

The binding affinities of peptides to LPS were examined using the BODIPY-TR-cadaverine fluorescent dye (BC, Sigma, United States) displacement assay, in which a probe bound to cell-free LPS results in dequenching of BC fluorescence. Stock solutions of LPS from *E. coli* O111:B4 (5 mg/mL; Sigma-Aldrich, China) and BC (2.5 mg/mL; Sigma-Aldrich, China) were prepared and diluted in Tris buffer (pH 7.4, 50 mM) to reach a final concentration of 50 μg/mL of LPS and 5 μg/mL BC. A 100 μL aliquot of the LPS-probe and peptide mixture (50 μL LPS and BC solutions at final concentrations of 25 μg/mL LPS and 2.5 μg/mL BODIPY-TR-cadaverine, 50 μL peptide with various concentrations ranging from 1 to 64 μM) was added in a sterile 96-well black plate. The fluorescence was measured on a spectrofluorophotometer (the Infinite 200 pro, Tecan, China) at excitation and emission wavelengths of 580 and 620 nm, respectively, with each test performed independently in triplicate. The values were converted to % Δ*F* (AU) using the following equation:

(4) $$\%\;\mathrm{\Delta F}(\mathrm{AU})=[(F_{\mathrm{obs}}-F_{0})/(F_{100}-F_{0})]\times 100$$

where *F*_obs_ is the observed fluorescence at a given peptide concentration, *F*_0_ is the initial fluorescence of BC with LPS in the absence of peptides, and *F*_100_ is the BC fluorescence with LPS cells upon addition of 10 μg/mL polymyxin B, which is a prototype LPS binder and was used as a positive control.

#### Scanning Electron Microscopy (SEM) Analysis

For SEM sample preparation, *E. coli* ATCC 25922 and *S. aureus* ATCC 29213 cells were collected at logarithmic phase, centrifuged at 1000 × *g* for 10 min and resuspended to an OD_600_ of 0.1–0.2 with 10 mM PBS. The peptides were added to achieve a final concentration of 1 × MICs and the mixed suspension was cultivated at 37°C for 1 h. Bacteria incubated without peptide served as the control. Next, the bacteria cells were centrifuged at 5000 × *g* for 5 min, washed with PBS three times and fixed with 2.5% (w:v) glutaraldehyde at 4°C overnight. The bacterial pellets were washed with PBS and dehydrated for 10 min with a series of graded ethanol solutions (50, 70, 90, and 100%). Following dehydration, the dried bacterial cells were transferred to a mixture (v:v = 1:1) of alcohol and tert-butanol for 30 min, followed by pure tert-butanol for 1 h. After drying using a critical point dryer, the bacterial specimens were coated and visualized using a field emission scanning electron microscope (Hitachi S-4800, Japan).

#### DNA Binding Assay

Gel retardation experiments were performed as previously described (Zhu et al., 2015). The total genomic DNA from *E. coli* was extracted using the rapid bacterial genomic DNA isolation kit (Sangon Biotech, China). First, increasing concentrations of peptides were mixed with 400 ng of genomic DNA in 20 μL of binding buffer [5% glycerol, 10 mM Tris-HCl (pH 8.0), 1 mM EDTA, 1 mM dithiothreitol, 20 mM KCl, and 50 μg/mL bovine serum albumin] and incubated at 37°C for 1 h. Next, 4 μL of native loading buffer [10% Ficoll 400, 10 mM Tris-HCl (pH 7.5), 50 mM EDTA, 0.25% bromophenol blue, and 0.25% xylene cyanol] was mixed with the samples and a 12 μL aliquot was subjected to 1% agarose gel electrophoresis in 0.5× Tris borate-EDTA buffer (45 mM Tris-borate and 1 mM EDTA, pH 8.0). Finally, gel retardation was visualized using UVP bioimaging systems.

### Statistical Analysis

The data were analyzed by ANOVA using the SPSS 18.0 software. Quantitative data are presented as the mean ± standard deviation. *P* < 0.01 was considered as statistically significant.

## Results

### Characterization of the Peptides

The structures and molecular weights of the peptides were verified by MALDI-TOF MS. All peptides had molecular weights consistent with their theoretical values, suggesting that the peptides were successfully synthesized. The relative hydrophobicity of the symmetric-end peptides was reliably reflected by their different HPLC retention times, and the hydrophobicity increased with increasing HPLC retention times (Lee et al., 2014; Zhu et al., 2014). The HPLC retention times for PP, PQ, GG, Qa and Qna were 11.409, 12.385, 10.24, 10.11, and 10.027 min, respectively, indicating the following hydrophobic order: PQ > PP > GG > Qa > Qna; these data showed that Qa has a similar mean hydrophobicity to Qna.

### Secondary Structure of the Peptides

**FIGURE 1:**
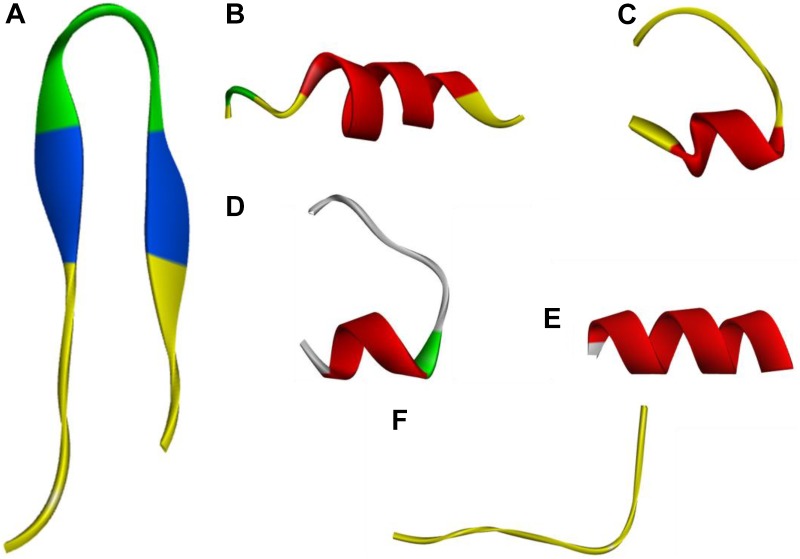
Three-dimensional structure projections of peptides by I-Tasser. **(A)**, PG-1; **(B)**, PQ; **(C)**, PP; **(D)**, GG; **(E)**, Qa; **(F)**, Qna.

Three-dimensional models of the peptides, obtained by I-Tasser, are shown in Figure 1. PQ, PP and Qa contained α-helical content, while PP and GG showed a mixture conformation of structures including α-helical and β-hairpin conformations. Qna showed a linear and unordered conformation. The control peptide PG-1 displayed a typical β-hairpin structure.

**FIGURE 2:**
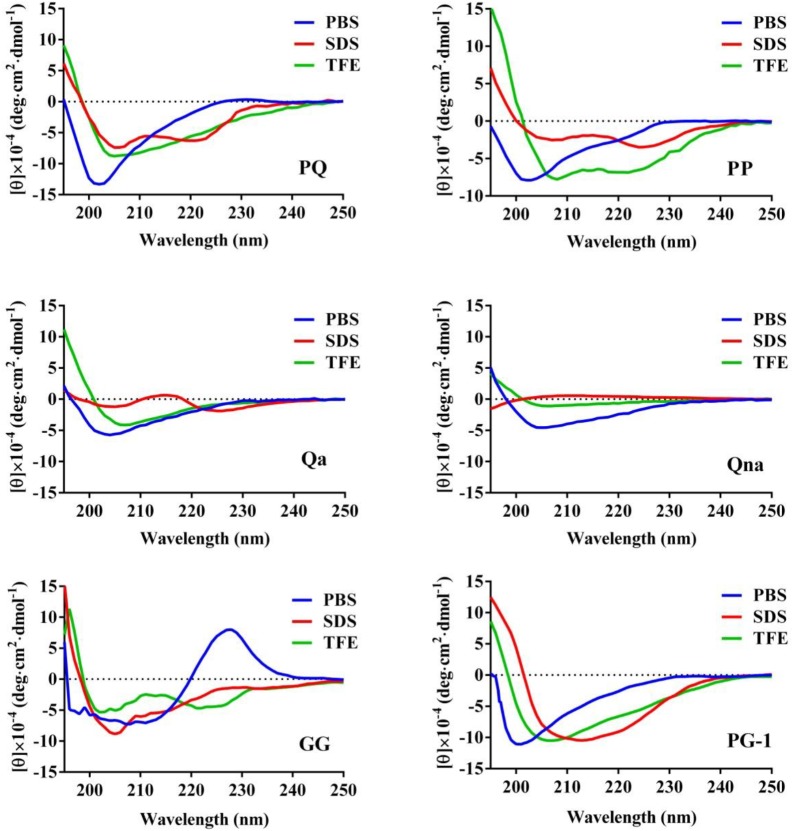
The CD spectra of the peptides. The peptides were dissolved in 10 mM sodium phosphate buffer (pH 7.4), 50% TFE and 30 mM SDS. The mean residue ellipticity was plotted against wavelength. The values from three scans were averaged per sample, and the peptide concentrations were fixed at 150 μM.

The CD spectroscopy was used to determine the secondary structures of the peptides in the membrane-mimetic environment. The spectra of the peptides in 10 mM PBS (pH = 7.4), 50% TFE and 30 mM SDS are shown in Figure 2. In 10 mM PBS (pH = 7.4), these peptides showed unordered conformations with a negative peak around 198 nm. In the 30 mM SDS and 50% TFE environments, PQ, PP, and GG demonstrated a slightly α-helical structure with positive peaks approximately 190 nm and negative peaks approximately 208 nm and 225 nm, while Qa and Qna showed unusual peaks, which correspond to a mixture of structures in the two membrane-mimetic environments. The percentage of different secondary structures for the designed peptides in 50% TFE and 30 mM SDS is listed in Table 2. The results showed that the PQ and PP peptides were inclined to form an α-helical structure.

**Table 2:** Percentage of different secondary structure of the peptides in various solutions.

Peptides	PBS		SDS		TFE	
			
	α-helix	β-strand	α-helix	β-strand	α-helix	β-strand
PQ	26.91	11.16	32.02	6.15	36.82	4.28
PP	12.35	16.30	9.86	11.56	37.54	2.54
GG	1.49	32.19	11.99	9.42	18.98	11.53
Qa	2.89	19.89	5.54	14.12	3.08	18.1
Qna	3.46	17.46	1.29	21.49	0.73	18.82


### Antimicrobial Activities of the Peptides

**FIGURE 3:**
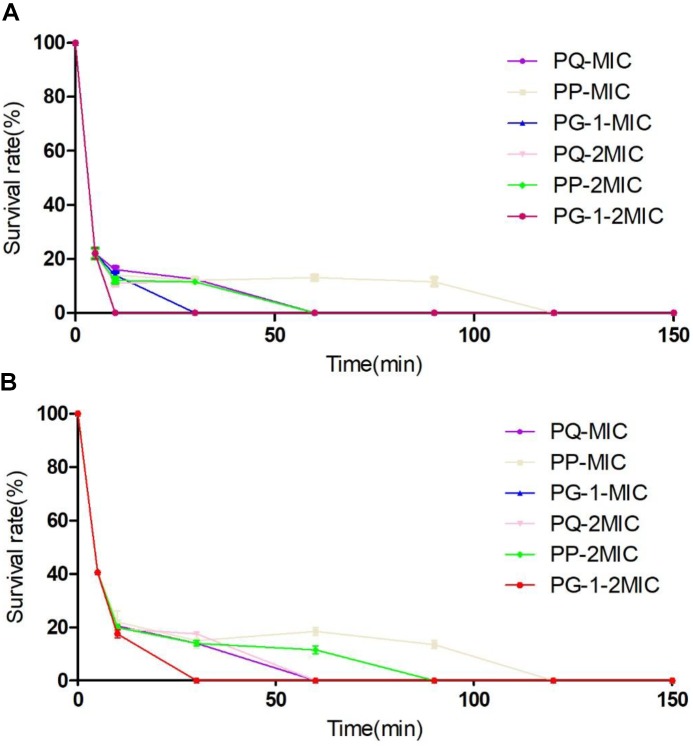
Time-kill kinetic curves of the peptides. Microbial survival of *Escherichia coli* **(A)** and *S. aureus* **(B)** after 5, 10, 30, 60, 90, 120, and 150 min of treatment with peptides at MIC and 2 × MIC.

The antimicrobial activities of the designed peptides against both Gram-negative and Gram-positive bacteria are summarized in Table 3. Compared with PQ with 18 amino acids, PP with 14 amino acids displayed higher antimicrobial activity across the bacterial species. Furthermore, PP showed approximately two-five-fold higher antimicrobial activity than the shorter peptides Qa and Qna, but the antimicrobial activity of PP was lower than PG-1, with a geometric mean of the MIC values of 8.0 and 5.7, respectively. To further analyze the antimicrobial activities of PP, PQ, and PG-1, the time killing assay was further analyzed. The results indicated that the killing kinetics of all of the investigated peptides were time-dependent (Figure 3). At the 1 × MIC and 2 × MIC concentrations, PG-1 killed *E. coli* in 10 min, PQ killed *E. coli* in 30 min, and PP killed *E. coli* in 90 min and 120 min, respectively. For Gram-positive bacteria, PG-1 killed *S. aureus* in 5 min and 10 min, respectively, PQ killed *S. aureus* in 30 min, and PP killed *S. aureus* in 90 min and 120 min, respectively.

**Table 3:** Minimum inhibitory concentrations (MIC), MHC, and TI of the peptides against Gram-negative and Gram-positive bacterial strains.

	MIC^a^ (μM)					
	
	PQ	PP	GG	Qa	Qna	PG-1
**Gram-negative bacteria**						
*E. coli ATCC25922*	16	8	8	16	32	2
*E. coli* UB1005	8	4	8	8	16	2
*B. pyocyaneum* ATCC 27853	8	4	16	16	32	16
*S. typhimurium* ATCC 7731	8	8	64	16	32	8
*S. pullorum* C79-13	8	4	8	16	32	2
**Gram-positive bacteria**						
*S. aureus* ATCC 29213	8	16	16	8	32	8
*S. aureus* ATCC 43300	8	16	32	16	32	2
*S. epidermidis* ATCC 12228	8	16	32	16	32	16
*S. faecalis* ATCC 29212	16	8	32	16	64	16
*B. subtilis* CMCC 63501	16	8	16	32	64	8
MHC^b^ (μM)	>128	>128	>128	>128	>128	2
GM^c^ (μM)	9.8	8.0	18.4	14.9	34.3	5.7
Therapeutic index^d^	26.1	32.0	13.9	17.2	7.5	0.4


*^*a*^Minimum inhibitory concentrations (MIC) were determined as the lowest concentration of the peptides that inhibited bacteria growth; ^*b*^minimum hemolysis concentration (MHC) was defined as the lowest peptide concentration that induces ≥5% hemolysis of human red blood cells (hRBC); ^*c*^GM denotes the geometric mean of MIC values from all microbial strains in this table; ^*d*^Therapeutic index (TI) is the ratio of the MHC to the geometric mean of MICs*.

### Evaluation of Toxicity

**FIGURE 4:**
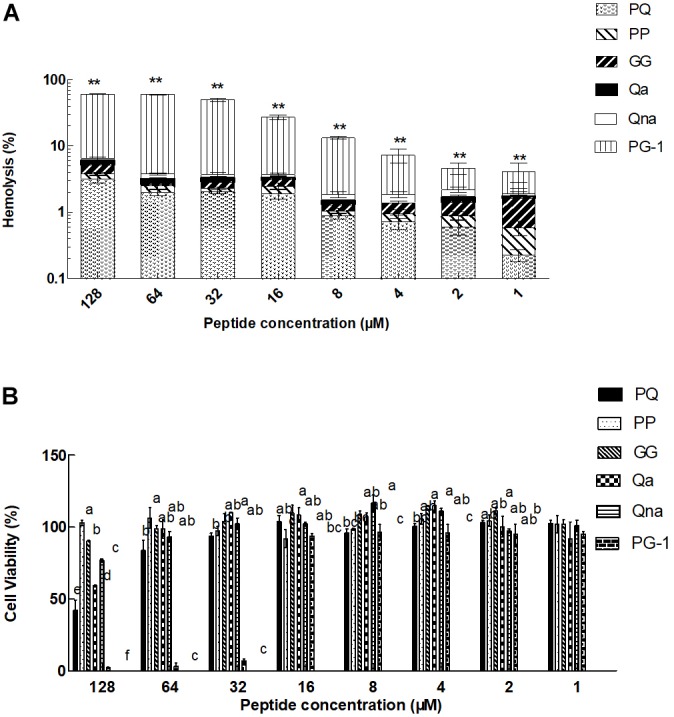
Eukaryotic cells toxicity of peptides against hRBCs **(A)** and IPEC-J2 cells **(B)**. *^∗∗^P <* 0.01, compared to values for PG-1 **(A)**. Means in the same concentration with different superscript indicate a very significant difference (*P* < 0.01) **(B)**. The graphs were derived from the average of three independent trials.

The toxicity of the peptides against hRBCs and IPEC-J2 cells was evaluated using serial peptide concentrations in the range from 0.5 to 128 μM, and the results are summarized in Table 3 and Figure 4. All the novel peptides had no toxicity against human erythrocytes, even at the highest concentration of 128 μM with hemolytic concentrations <5%. PP exhibited no cytotoxicity against IPEC-J2 cells, with a more than 99% cell survival rate even at the 128 μM concentration. The PP peptide was the best of the five peptides tested, with the highest cell selectivity toward bacterial cells over eukaryotic cells, implying a wider therapeutic window.

### Salt Sensitivity Assays

To investigate salt sensitivity, the antimicrobial activities of PP and PQ were tested after the addition of physiological concentrations of different salts (Table 4). The antimicrobial activities of PP and PQ against *E. coli* ATCC 25922 were promoted or maintained by the presence of all the tested cations except Ca^2+^, which decreased the antimicrobial activity of PQ by increasing the MIC value from 16 to 32 μM. Compared with the activity against *E. coli* ATCC 25922, the antimicrobial activity of PP against the Gram-positive bacterium *S. aureus* ATCC 29213 was decreased by the presence of monovalent Na^+^, which made PP lose almost all its antimicrobial activity, and divalent Ca^2+^, which decreased the antimicrobial activity of PP by increasing the MIC value from 16 to 32 μM.

**Table 4:** Minimum inhibitory concentrations values of peptides in the presence of physiological salts^a^.

Peptides	MIC^a^ (μM)							
	
	Control	NaCl^b^	KCl^b^	NH_4_Cl^b^	MgCl_2_^b^	CaCl_2_^b^	ZnCl_2_^b^	FeCl_3_^b^
**Gram-negative strain**								
***E. coli* ATCC 25922**								
PQ	16	8	4	4	4	32	4	8
PP	8	8	4	2	2	4	2	4
PG-1	2	4	2	2	2	4	2	4
**Gram-positive strain**								
***S. aureus* ATCC 29213**								
PQ	8	8	8	8	8	8	8	8
PP	16	128	16	8	16	32	4	8
PG-1	8	32	8	8	8	16	8	8


*^*a*^Minimum inhibitory concentrations (MIC) were determined as the lowest concentration of the peptides that inhibited bacteria growth. ^*b*^The final concentrations of NaCl, KCl, NH_*4*_Cl, MgCl_*2*_, CaCl_*2*_, ZnCl_*2*_, and FeCl_*3*_ were 150 mM, 4.5 mM, 6 μM, 1 mM, 2 mM, 8 μM, and 4 μM, respectively, and the control MIC values were determined in the absence of these physiological salts*.

### Serum Stability

**FIGURE 5:**
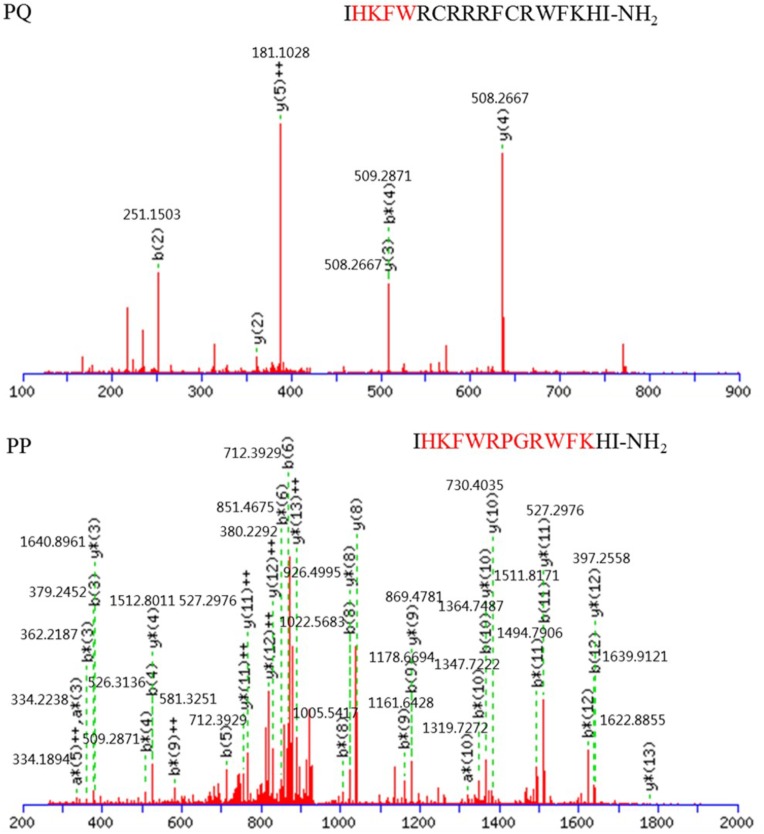
MS/MS spectra of precursor ions that correspond to peptides PQ and PP. Red font is reserved peptide.

In addition to salt sensitivity, the serum sensitivity against the Gram-negative bacteria *E. coli* ATCC 25922 and Gram-positive *S. aureus* ATCC 29213 was also investigated in the presence of 25 or 50% serum (Table 5). The MICs for all the tested peptides were increased in the presence of 25 or 50% serum. PQ was more tolerant to serum against the Gram-negative bacteria *E. coli* ATCC 25922 and Gram-positive *S. aureus* ATCC 29213 compared with PG-1 and PP. Additionally, the activity of PP against the Gram-positive *S. aureus* ATCC 29213 was mostly lost in the presence of 50% serum. Using the LC-MS/MS test, the AMPs PP and PQ were not stable in the presence of 50% serum, and their amino acid sequences were destroyed (Figure 5).

**Table 5:** Minimum inhibitory concentrations values of peptides in the presence serum and heat.

Peptides	MIC^a^ (μM)			
	
	Control	50% serum	25% serum	Heat (100°C)
**Gram-negative strain *E. coli* ATCC 25922**				
PQ	16	32	32	32
PP	8	64	32	8
PG-1	2	64	64	8
**Gram-positive strain *S. aureus* ATCC 29213**				
PQ	8	16	8	32
PP	16	128	32	16
PG-1	8	16	16	16


*^*a*^Minimum inhibitory concentrations (MIC) were determined as the lowest concentration of the peptides that inhibited bacteria growth*.

### Thermal Stability

The results of the antimicrobial activities of PP and PQ against the Gram-negative bacteria *E. coli* ATCC 25922 and Gram-positive *S. aureus* ATCC 29213 upon heating are also shown in Table 5. PP exhibited strong thermal stability by retaining MICs against *E. coli* and *S. aureus*, though incubation at 100°C decreased the antimicrobial activities of PQ and PG-1.

### The Effects of pH on the Antimicrobial Activity of the Peptides

Mueller–Hinton broth was adjusted with HCl or NaOH to investigate the effects of pH (4.0–10.0) on antibacterial activity. The results for the antimicrobial activities of PP and PQ against the Gram-negative bacteria *E. coli* ATCC 25922 and Gram-positive *S. aureus* ATCC 29213 was also shown in Table 6. The results showed that PP, PQ and PG-1 could maintain their antimicrobial activities at different pH values.

**Table 6:** Minimum inhibitory concentrations values of peptides in different pH values.

Peptides	MIC^a^ (μM)			
	
	pH 4.0	pH 6.0	pH 8.0	pH 10.0
**Gram-negative strain *E. coli* ATCC 25922**				
PQ	8	8	4	4
PP	8	8	16	8
PG-1	4	4	4	4
**Gram-positive strain *S. aureus* ATCC 29213**				
PQ	8	8	8	8
PP	16	16	16	16
PG-1	8	8	8	16


*^*a*^Minimum inhibitory concentrations (MIC) were determined as the lowest concentration of the peptides that inhibited bacteria growth*.

### Antimicrobial Mechanism Study

**FIGURE 6:**
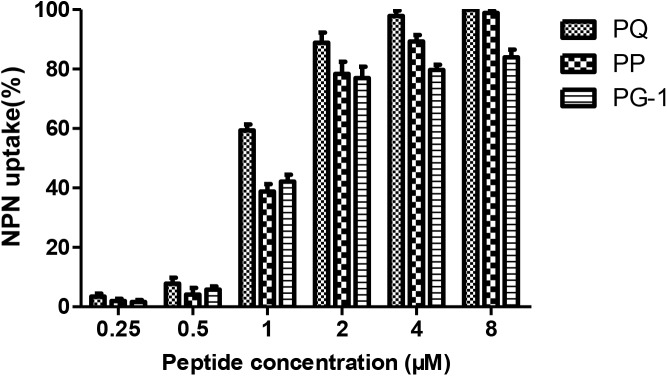
Outer membrane permeabilization assays of PQ, PP and PG-1.

Numerous antibacterial peptides kill bacteria predominantly via membrane permeabilization and subsequent structural disruption. Thus, the ability of peptides to permeabilize the outer membrane was tested first using a NPN uptake assay. As shown in Figure 6, the three peptides were able to permeabilize the outer membrane of Gram-negative bacteria at concentrations ranging from 0.5 to 8 μM, which followed a concentration-dependent increase in the response. In the presence of 2 μM peptide, the outer membrane permeability of the two novel peptides was greater than 70%. Moreover, at concentrations ranging from 2 to 8 μM, the outer membrane permeability of the two novel peptides was higher than that of PG-1.

**FIGURE 7:**
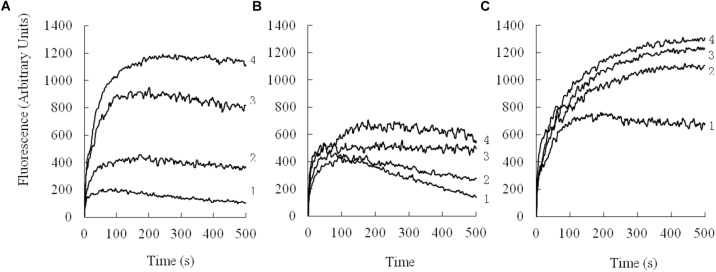
Cytoplasmic membrane depolarization of *E. coli* UB1005 was measured by using the membrane potential-sensitive dye, DiSC3-5. **(A)**, PQ; **(B)**, PP; **(C)**, PG-1. 1, 2 μM; 2, 4 μM; 3, 8 μM; 4, 16 μM.

Depolarization of the cytoplasmic membrane on *E. coli* was assessed by using a membrane potential-dependent probe (diSC3-5) that is quenched by the cytoplasmic membrane. As shown in Figure 7, the depolarization by different concentrations of peptides was monitored over a period of 500 s. All the peptides depolarized the bacterial cytoplasmic membrane in a dose- and time-dependent manner.

**FIGURE 8:**
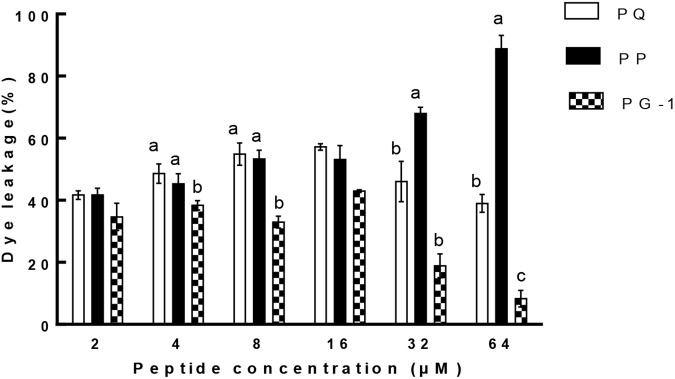
Percent leakage of the dye calcein from negatively charged LUVs in the presence of the peptides. The graphs were derived from average of three independent trials. Means in the same concentration with different superscript indicate a very significant difference (*P* < 0.01).

Liposome leakage assay is usually conducted to evaluate whether the peptides exert antimicrobial activities by pore formation and/or membrane perturbation. We measured their abilities to induce calcein leakage from negatively charged LUVs (bacterial membrane-mimicking environment). The results (Figure 8) showed that PP induced a significantly higher leakage activity than other two tested peptides above 32 μM (*P* < 0.01). PP and PQ exhibited more than 40% dye leakage at all tested concentrations, indicating the destruction of liposomes by pore formation.

**FIGURE 9:**
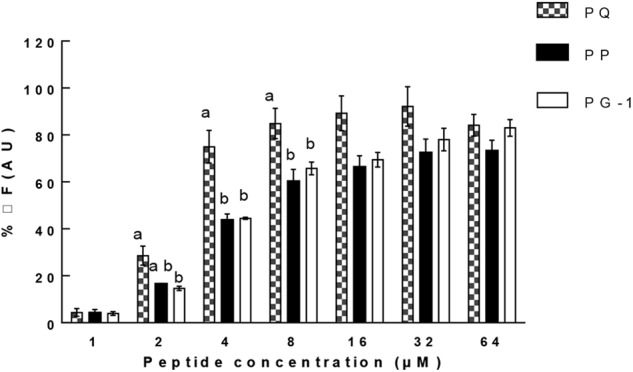
Peptide binding affinity to LPS from *E. coli* 0111: B4. The graphs were derived from average of three independent trials. Means in the same concentration with different superscript indicate a very significant difference (*P* < 0.01).

In addition to the membrane disruption assays, we also examined the ability of PP and PQ to bind to LPS with a fluorescence-based displacement assay with BC because LPS is the major constituent of the outer membrane on Gram-negative bacteria. The results (Figure 9) showed that all the tested peptides produced a strong dose-dependent enhancement of fluorescence, showing the binding of these peptides to LPS. Furthermore, PQ generated fluorescence above 80% at the MIC concentration, while other peptides were less than 60% at their MICs.

**FIGURE 10:**
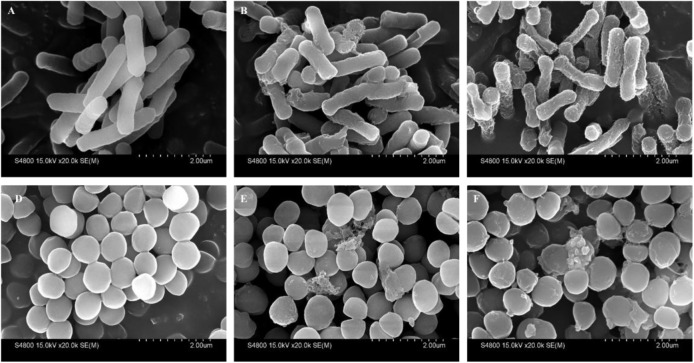
Scanning electron micrographs of *E. coli* 25922 and *S. aureus* 29213 treated with PP and PQ. SEM micrographs of *E. coli*: **(A)** Control, no peptides; **(B)** PQ-treated, 16 μM; **(C)** PP-treated, 8 μM. SEM micrographs of *S. aureus*: **(D)** Control, no peptides; **(E)** PQ-treated, 8 μM; **(F)** PP-treated, 16 μM. Bacteria in mid-logarithmic growth were treated with peptides at 1× MIC for 1 h.

The cell morphology and membrane integrity upon treatment with peptides were directly observed by SEM (Figure 10). These images showed that the control *E. coli* and *S. aureus* cells had a bright and smooth surface, but the membrane surface of the *E. coli* cells treated with PP and PQ became completely roughened and corrugated and even induced atrophy and fracture. The effects of these peptides on *S. aureus* showed that PP and PQ could induce obvious blebbing of the cell envelope or even induce fracture.

**FIGURE 11:**
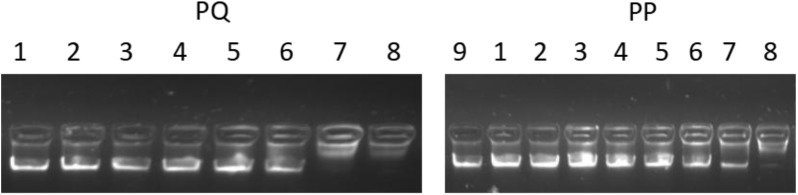
The total genomic DNA binding assay. Various concentrations of peptides were incubated with 600 ng DNA for 1 h at room temperature; Lane 1: genomic DNA alone; lane 2: with 2 μM peptide; lane 3: with 4 μM peptide; lane 4: with 8 μM peptide; lane 5: with 16 μM peptide; lane 6: with 32 μM peptide; lane 7: with 64 μM peptide; lane 8: with 128 μM peptide; lane 9: with 256 μM peptide.

In addition to the membrane interaction assays, a DNA-binding assay was adopted to investigate the possibility of intracellular effects. As shown in Figure 11, PP and PQ exhibited DNA-binding abilities above 256 and 128 μM, respectively.

## Discussion

In this study, we hybridized α-helix and β-turn sequence-motifs in a symmetric-end template to take advantage of different particular activities together. Additionally, the symmetric sequence without the β-turn and sequence with imperfectly amphipathic motifs was also synthesized to investigate the influence of these factors. The secondary structure proclivities of these peptides in both water and membrane-like environments were determined. Considering the results from the three-dimensional structure models, CD spectroscopy and percentage of different secondary structures of the peptides, the designed peptides PQ and PP with the α-helix and β-turn sequence-motifs exhibited a tendency for an α-helical structure in 30 mM SDS (environment compared to a negatively charged prokaryotic membrane) and a secondary structure with α-helical peptides with a central β-turn in 50% (v/v) TFE (mimicking the hydrophobic environment of the microbial membrane) (Shao et al., 2018).

The results from the direct antimicrobial activity assays demonstrated that all these peptides possessed broad antimicrobial activities against both Gram-negative and Gram-positive bacteria, including methicillin-resistant *S. aureus* ATCC 43300. PP has higher activity than PQ, which was most likely due to the appropriate net positive charge. As observed in previous reports, an appropriate net charge is crucial for antimicrobial activity, and peptides with a +6 ∼ +7 charge may be optimal, as increasing the positive charges does not correlate with increased antimicrobial activity or even decreased activity (Yin et al., 2012; Liu et al., 2013). The results showing that PP had higher activity than Qa, suggesting that the addition of the β-turn sequence-motif might improve the antimicrobial activity. Additionally, Qa was more efficient than Qna, most likely due to the perfect amphipathicity of the last. The result from the time killing assay was in accordance with the observation that higher hydrophobicity and longer length appeared to strongly facilitate rapid peptide approach and insertion into the bacterial lipid bilayer resulting in cell death (Gopal et al., 2013). The three-dimensional results showed that Qa had the potential to form a more ideal amphiphilic α-helical structure than Qna. This result was consistent with previous results indicating that the propensity for α-helix formation and amphipathicity might be some of the most important factors in bacterial cell death (Ma et al., 2015). The result that the activities of PP and PQ were better than Qa and Qna was most likely due to the addition of β-turn sequence-motif, which might promote the tightly hydrophobic core and improve the antimicrobial activity (Chou et al., 2016).

It is widely known that biocompatibility is essential for an *in vivo* application; for example, PG-1, which showed equal MIC and MHC values, was ineffective in the *in vivo* application. To demonstrate the biocompatibility of these novel peptides, the cytotoxic effects of the peptides have been detected in this study. At all the tested concentrations, the novel peptides had significantly lower hemolytic activities than the typical β-hairpin peptide PG-1, showing improved selectivity toward the anionic components of microbial cell membranes, which might be due to the short length of these novel peptides, as previous studies found that cell selectivity was decreased with increasing peptide chain length (Niidome et al., 2005; Liu et al., 2007). It was further confirmed that PP exhibited no cytotoxicity against IPEC-J2 cells, with a greater than 99% cell survival rate even at the 128 μM concentration. Thus, this short sequence structure showed high therapeutic value.

In addition to the cell selectivity of the AMPs, another important problem for their pharmaceutical development is stability. The addition of cosolvents to peptide solutions may lead to a variety of adverse impacts such as denaturation or decreased activity. To demonstrate the stability of these peptides, we investigated the effects of salts, serum, heat and pH on the antimicrobial activities of the optimized peptides. Interestingly, the antimicrobial activities of PP and PQ were only slightly compromised, or even increased, under salt conditions. It was illustrated that the interaction of AMPs with bacterial membranes depends on the establishment of attractive electrostatic forces between the positive peptide and the negative membrane (Dashper et al., 2005; Huang et al., 2011; Xu et al., 2014). The increased PP and PQ activity against *E. coli* in the presence of some salts might due to the addition of the β-turn sequence-motif, because the β-turn conformation may increase the salt sensitivity due to the increased hydrogen bonding and hydrophobic interactions (Chou et al., 2016). Additionally, the report that the stability of PG-1 and its analogs was related to the salt sensitivity also demonstrated that the addition of the β-turn sequence-motif was beneficial for improving the resistance to salt conditions (Lai et al., 2002). This result showing that PQ had slightly higher antimicrobial activity than PP in the presence of physiological salts, was in accordance with the higher net positive charge of PQ that may be more tolerant to divalent cations (Aquila et al., 2013). The decreased activity of PP in the presence of Na^+^ against *S. aureus* might be caused by hindering the electrostatic interaction between the peptides and membranes, which may prevent peptides from entering the bacterial membrane and result in decreased AMP antimicrobial activity. The decreased activities of the peptides in the presence of salts are in accordance with the idea that free ions in the surrounding media often decrease the binding efficacy of the peptides and finally impair their killing efficiency. The result that PP and PQ retained partial antimicrobial activity after treatment with serum and at different pH values demonstrated that PP and PQ might display moderate stability in the *in vivo* environment. However, PP and PQ were unstable in human serum via HPLC and MS analyses. Additionally, because many procedures such as food or feed processing involve a heating step, the heat stability of these peptides is an important characteristic to ascertain their potential application (Ma et al., 2012). These studies demonstrated that although some shortcomings exist, this pattern is a potential candidate for antimicrobial agents in clinical therapy.

It has been indicated that α-helical peptides exerted antimicrobial activity via membrane destruction (Huang, 2000; Ting et al., 2014); thus, we investigated the effects of PP and PQ on the membrane via membrane permeability and microscopic techniques. Since membrane depolarization is a crucial characteristic of peptide-membrane interactions, we determined the antibacterial potency of AMPs based on the outer membrane permeability and cytoplasmic membrane electrical potential assays. The results of a concentration-dependent increase in the outer membrane permeability and conspicuous cytoplasmic membrane depolarization suggested that these peptides might exert antimicrobial activities via bacterial membrane permeabilization. Additionally, the SEM study further confirmed that the peptides caused damage to the membranes. These results indicated that the first step in bacteriostasis is that cationic peptides selectively bind the anionic lipids of the bacterial outer membrane and then insert into the hydrophobic core of the membrane bilayer, eventually disrupting the bilayer integrity, which is in accordance with previous reports (Sani and Separovic, 2016; Wenzel et al., 2016).

To better understand the membrane-active mechanism, the outer leaflet of the outer membrane, which is mainly composed of LPS, was tested for LPS binding. A previous study showed that cationic AMPs can interact with the anionic amphiphilic lipid via hydrophobic interactions between the LPS alkyl chains and non-polar amino acid side chains (Ong et al., 2013). Thus, it was not surprising that PQ, which has a stronger hydrophobicity and more positive charge, showed the highest LPS-binding ability among the three tested peptides.

In addition to their membrane-active mechanism, it is known that AMPs can lead to cellular inactivation by binding intracellular targets (e.g., DNA) (Teixeira et al., 2012). The present result showed that the peptide bound DNA at much higher concentrations than MICs, which demonstrated that the peptides could not kill the bacteria at MIC values by DNA binding. This suggested that PP and PQ exerted their antimicrobial actions by damaging the cell membrane through pore formation, inducing membrane atrophy and fracture due to the leakage of intracellular contents, and further developed antimicrobial activity by traversing the bacterial membrane and binding DNA.

In this study, we report two peptides that were designed based on the hybridization of and α-helix and β-turn in a symmetric-end template. The embedding of the β-turn sequence-motif in the symmetric-end template improved the antimicrobial activity against both Gram-negative and Gram-positive bacteria, including methicillin-resistant *S. aureus* ATCC 43300, with selectivity increased and condition sensitivity decreased. Furthermore, the mechanistic assays indicated that PP and PQ kill bacterial cells through membrane pore formation, which leads to leakage of the cytosol. Our results indicate that PP and PQ are potential candidates for *in vivo* studies without considerably changing the antimicrobial activity.

## Ethics Statement

All procedures performed in studies involving human participant (Zhihua Wang) were in accordance with the ethical standards of the institutional and/or national research committee and with the Helsinki Declaration and its later amendments or comparable ethical standards.

## Author Contributions

ND and SC conceived and performed most of the experiments, including circular dichroism spectroscopy, the antimicrobial assay, the hemolytic assay, the membrane permeability assay and so on. JL and CX performed condition sensitivity assays. XL and BC performed scanning electron microscopy (SEM) analysis. AS and LX reviewed and edited the manuscript. In the process of revising manuscript, LX performed supplementary serum stability experiment (HPLC and LC-MS/MS).

## Conflict of Interest Statement

The authors declare that the research was conducted in the absence of any commercial or financial relationships that could be construed as a potential conflict of interest.

## Footnotes

1 http://www.expasy.org/tools/protparam.html

2 http://cbdm-01.zdv.uni-mainz.de/~andrade/k2d2//index.html

3 http://zhanglab.ccmb.med.umich.edu/I-TASSER/

## Supplementary Material

DATA SHEET S1 RP/HPLC elution profile of the peptides.
