# Supplementary material for: Short Symmetric-End Antimicrobial Peptides Centered on β-Turn Amino Acids Unit Improve Selectivity and Stability
Source: Front Microbiol. 2018 Nov 27;9:2832. doi: 10.3389/fmicb.2018.02832 (PMC6277555; doi:10.3389/fmicb.2018.02832)
Supplement: DATA SHEET S1 — RP/HPLC elution profile of the peptides. [file Data_Sheet_1.pdf]

HPLC Report

Structure : PG-1 RR-18
Number : 010250036
Lot No : P150506-SX451928
Column : 4.6×250mm,Boston Green ODS-AQ
Solvent A : 0.1% trifluoroacetic in 100% acetonitrile
Solvent B : 0.1% trifluoroacetic in 100% water
Gradient :

	A	B
0.01min	10%	90%
25min	50%	50%
25.1min	100%	0%
30min	STOP	

Flow rate : 1.0 mL/min
Wavelength : 220nm
Volume : 5ul

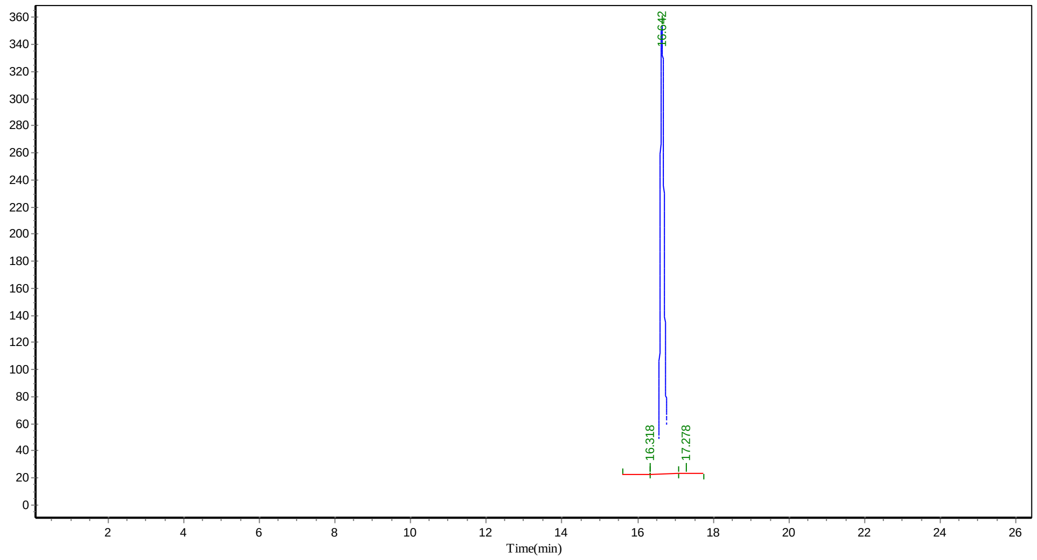

Peak No.	Ret Time	Height	Area	Conc.
1	16.318	1669.018	36819.746	1.3623
2	16.642	332062.969	2637148.250	97.5742
3	17.278	1315.297	28741.291	1.0634
**Total**				**100.0000**

Product Name:PQ II-18

Instrument No: 0200023

Lot No :P151224-SL490368

Column :4.6*250mm,Kromasil C18-5

Solvent A :0.1%Trifluoroacetic in 100% Acetonirile

Solvent B :0.1%Trifluoroacetic in 100% Water

Gradient :		A	B
	0.01min	22%	78%
	25min	47%	59%
	25.1min	100%	0%
	30min	Stop	

Flow rate :1.0ml/min

Wavelength :220nm

Volume:10ul

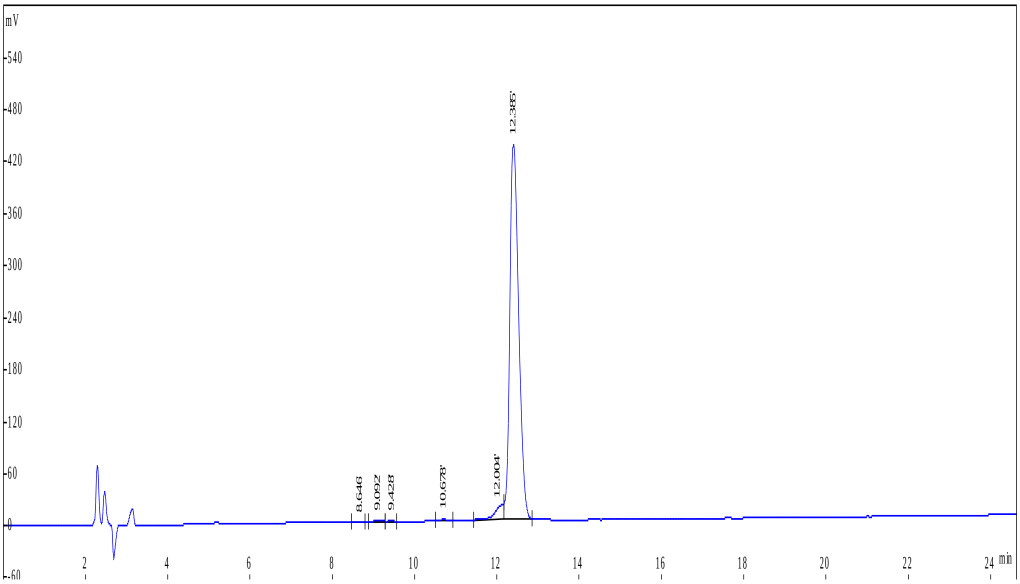

Rank	Time	Conc.	Area	Height
1	8.646	0.0878	5593	803
2	9.092	0.2298	14643	1193
3	9.428	0.0640	4078	653
4	10.678	0.2967	18905	1670
5	12.004	4.1678	265537	13406
6	12.385	95.1539	6062440	431700
Total		100	6371196	449425

Product Name:PP II-14

Instrument No: 0200023

Lot No :P151224-SL490369

Column :4.6*250mm,Kromasil C18-5

Solvent A :0.1%Trifluoroacetic in 100% Acetonirile

Solvent B :0.1%Trifluoroacetic in 100% Water

Gradient :	A	B
0.01min	20%	80%
25min	45%	55%
25.1min	100%	0%
30min	Stop	

Flow rate :1.0ml/min

Wavelength :220nm

Volume :10ul

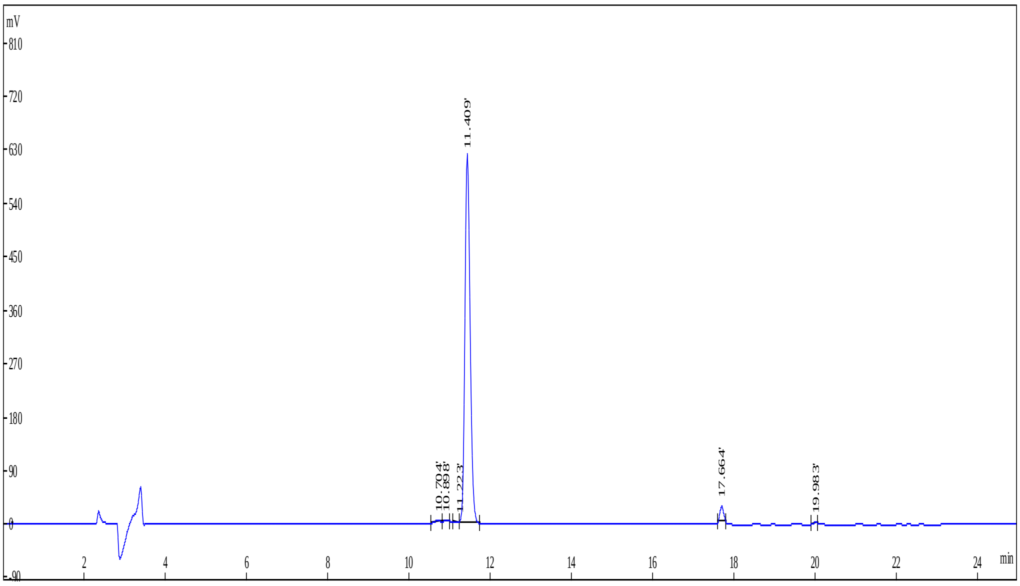

Rank	Time	Conc.	Area	Height
1	10.704	0.5635	32615	3331
2	10.898	0.4604	26650	3523
3	11.223	0.1355	7844	2205
4	11.409	95.2778	5514819	624401
5	17.664	3.0838	178497	28379
6	19.983	0.4790	27728	5153
Total		100	5788153	666992

Product Name:Qa II-12

Instrument No: 0200023

Lot No :P151224-SL490371

Column :4.6*250mm,Kromasil C18-5

Solvent A :0.1%Trifluoroacetic in 100% Acetonirile

Solvent B :0.1%Trifluoroacetic in 100% Water

Gradient :	A	B
0.01min	22%	78%
25min	47%	53%
25.1min	100%	0%
30min	Stop	

Flow rate :1.0ml/min

Wavelength :220nm

Volume :10ul

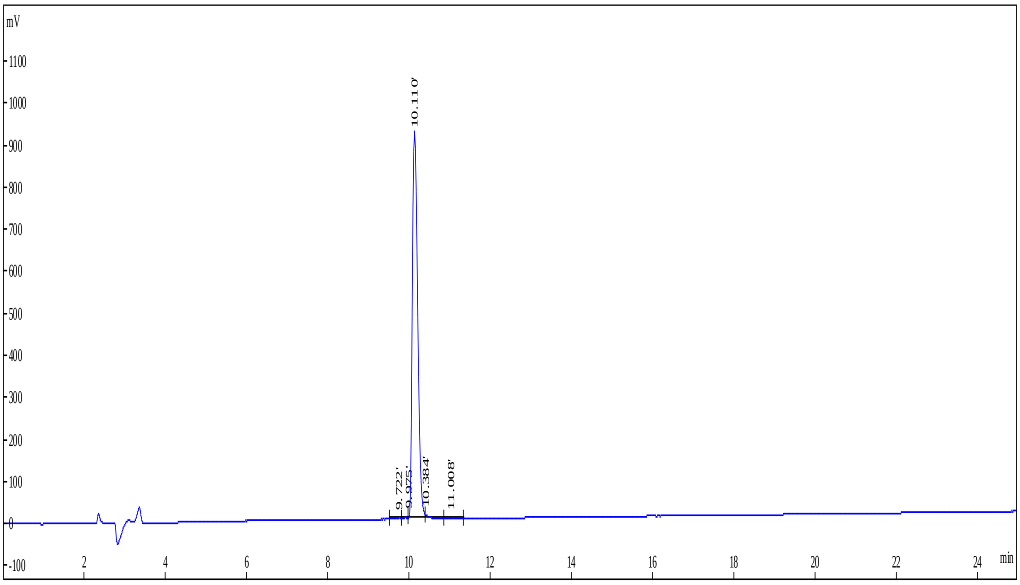

Rank	Time	Conc.	Area	Height
1	9.722	0.3561	30425	3429
2	9.975	0.3199	27333	4117
3	10.110	98.3617	8404918	923096
4	10.384	0.7147	61073	7885
5	11.008	0.2476	21160	1440
Total		100	8544909	939967

Product Name:Qna II-12

Instrument No: 0200023

Lot No :P151224-SL490372

Column :4.6*250mm,Kromasil C18-5

Solvent A :0.1%Trifluoroacetic in 100% Acetonirile

Solvent B :0.1%Trifluoroacetic in 100% Water

Gradient :	A	B
0.01min	25%	75%
25min	50%	50%
25.1min	100%	0%
30min	Stop	

Flow rate :1.0ml/min

Wavelength :220nm

Volume :10ul

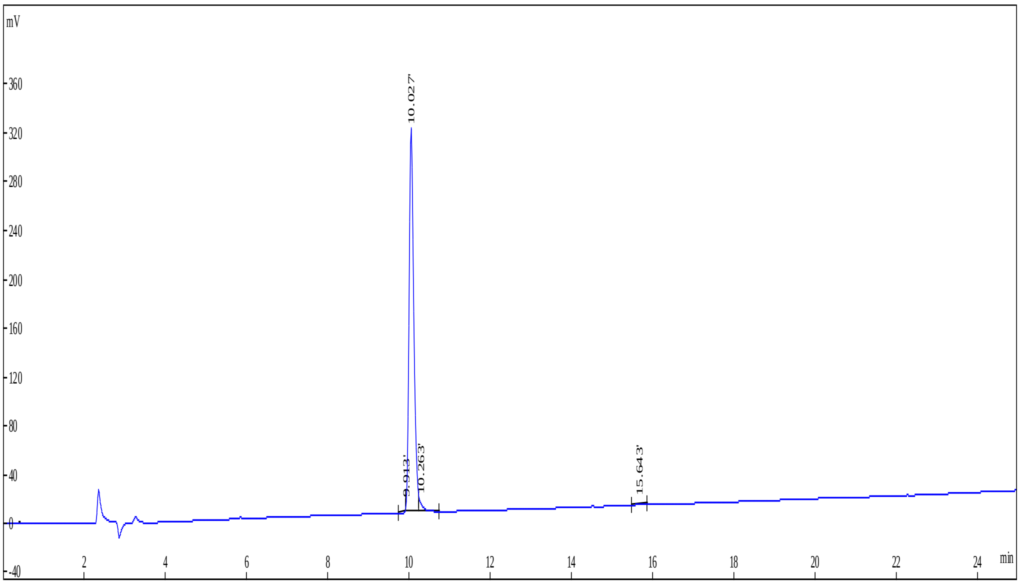

Rank	Time	Conc.	Area	Height
1	9.913	0.3904	10211	11048
2	10.027	97.0246	2537614	318590
3	10.263	2.1639	56595	6965
4	15.643	0.4211	11012	854
Total		100	2615432	337457
